# 

**Published:** 1889-10

**Authors:** 


					﻿io cts. a copy.
OCTOBEE, 1889.
$ 1.00 per year.
HALES*
,/AKWI-
HEALTH
ESTABLISHED 18t)4
Vi- 36
’c^-10,
OFFIOE: 206 BROADWAY, EVENING POST BUILDING, Room 11. N. Y.
Entered as Second-class Matter at the Post-Office, New York.
				

